# Pangenome Analysis of *Helicobacter pylori* Isolates from Selected Areas of Africa Indicated Diverse Antibiotic Resistance and Virulence Genes

**DOI:** 10.1155/2024/5536117

**Published:** 2024-03-04

**Authors:** Biigba Yakubu, Edwin Moses Appiah, Andrews Frimpong Adu

**Affiliations:** Department of Biochemistry and Biotechnology, Kwame Nkrumah University of Science and Technology, Kumasi, Ghana

## Abstract

The challenge facing Helicobacter pylori (H. pylori) infection management in some parts of Africa is the evolution of drug-resistant species, the lack of gold standard in diagnostic methods, and the ineffectiveness of current vaccines against the bacteria. It is being established that even though clinical consequences linked to the bacteria vary geographically, there is rather a generic approach to treatment. This situation has remained problematic in the successful fight against the bacteria in parts of Africa. As a result, this study compared the genomes of selected H. pylori isolates from selected areas of Africa and evaluated their virulence and antibiotic drug resistance, those that are highly pathogenic and are associated with specific clinical outcomes and those that are less virulent and rarely associated with clinical outcomes. 146 genomes of H. pylori isolated from selected locations of Africa were sampled, and bioinformatic tools such as Abricate, CARD RGI, MLST, Prokka, Roary, Phandango, Google Sheets, and iTOLS were used to compare the isolates and their antibiotic resistance or susceptibility. Over 20 k virulence and AMR genes were observed. About 95% of the isolates were genetically diverse, 90% of the isolates harbored shell genes, and 50% harbored cloud and core genes. Some isolates did not retain the cagA and vacA genes. Clarithromycin, metronidazole, amoxicillin, and tinidazole were resistant to most AMR genes (vacA, cagA, oip, and bab). *Conclusion*. This study found both virulence and AMR genes in all H. pylori strains in all the selected geographies around Africa with differing quantities. MLST, Pangenome, and ORF analyses showed disparities among the isolates. This in general could imply diversities in terms of genetics, evolution, and protein production. Therefore, generic administration of antibiotics such as clarithromycin, amoxicillin, and erythromycin as treatment methods in the African subregion could be contributing to the spread of the bacterium's antibiotic resistance.

## 1. Background

*Helicobacter pylori* (*H. pylori*) is one of the most studied infectious bacteria worldwide with 4.4 billion people estimated to be infected as of 2019. The bacterium is a spiral microaerophilic pathogen that returns a Gram-negative result on the Gram stain test. It intrudes the stomach mucoid lining with the aid of its helix appendages and thereby establishes infection of the gastrointestinal tract leading to the mirage of sicknesses [1–4]. It is usually acquired in the juvenile stages of life through a transmission yet unknown leading to the initiation of inflammations [5], which could last a lifetime if not treated.

Genomically, the bacteria consist of a huge family of diverse strains [6], and some of their genomes have been completely sequenced and explored. The genome is about 1.65 Mbps with average GC content of 38.9%, and an average of 1637 protein-coding genes [7]. These genes are not only heterogeneously distributed but also exert varying degrees of virulence in the host [8]. In humans, these genes are associated with diseases like gastritis and peptic ulcers, predicates of gastric cancers [9]. As a result, the pathogen has been considered by WHO as a disturbing class one carcinogen worldwide.

Infections of the bacteria in Africa continue to soar high with the continental average infection rate at 90% with Nigeria at 89%, Ghana at 75%, and Egypt at 95% [10]. The rates are also high in some low socioeconomic places such as East Asia. These rates have triggered questions as regards the efficacies and reliabilities of the treatment strategies adopted so far in the fight against the pathogen. In many instances, patients from various clinics diagnosed with *H. pylori* showed diverse gastro-related illnesses, including peptic ulcers, stomach tumors, mucosa-associated lymphoid tissue (MALT) lymphoma, and biliary tract cancer [11]. Apart from that, *H. pylori* infections have also led to the development of other gastric diseases, such as ischemic heart diseases [12], anemia, type 2 diabetes mellitus, insulin resistance, and adverse metabolic traits in individuals.

Weirdly, in the vast majority of infections that are recorded in Africa, a small percentage (10-20%) of the people suffer diseases [13], implying that the bacteria could be present in the human body system without inducing any sickness. What is unclear are the factors which trigger the pathogenesis in the select few as against the majority who remain periodic carriers of the bacterium. On the contrary, there is also proven evidence that *H. pylori* may be beneficial in human health [14] but in rather rare occasions. Other studies have corroborated Bravo's findings that the decreasing incidence of *H. pylori* infections in the developing world is paralleled by an increase in the incidence of allergies and autoimmune diseases [15] and the absence of *H. pylori* has also been linked to heightened incidence of diseases, such as celiac disease and multiple sclerosis [16]. However, what is unproven are the factors responsible for this inconsistent initiation of diseases.

Apart from the high infection rates in Africa, the bacterium is also known for its virulent factors which aid its disease initiation. The very common ones are the secretory systems (T4SS) and the *cagA* oncoproteins which have been linked to inflammations and cancer development of gastrointestinal tract of victims [17]. The bacteria initiate this virulence by invading the host and initiate diseases in stages. In the first stage, the bacteria's urease enzyme counterbalances the gastric pH of the host, enabling the occupation of gastric epithelial cells in the mucus layer [18]. The second stage is the bond of the bacteria to the stomach epithelium, which is believed to be enabled by its *babA* gene and *sabA* adhesins, permitting the injection of the virulent factors *cagA* [19] into the host cells, which leads to a strong universal resistant response and swelling of the stomach mucosa.

Other related virulent factors are *interleukin 8* and a neglected virulent factor duodenal ulcer promoting gene a, *dupA.* While the *IL-8* induces its *bab*A and *bab*B genes [20], the *dupA* is highly linked with duodenal ulcer development and reduces risk of gastric cancer [21]. These genes facilitate the attachment of the bacterium to exact human blood group Lewis's antigens, which is connected with various disease consequences. Likewise, the epithelium antigen in *H. pylori*, which is triggered on interaction with the epithelium, is also revealed to persuade high levels of IL-8 [22]. Hence, strains with *OipA* gene in its lively forms as *iceA* and *babA*, which are *cagA+* and *vacA s1*, have been shown to cause a more terrifying outcome in selected cases [23]. What is fundamentally missing are the specific strains, which express these genes and the specific environment that enhances their respective expressions or virulence.

In summary, there is class-wide reminiscence of variability in *H. pylori* bacterium's virulence which ought to be investigated. As a result, we aim to establish the comparative presence of these virulence and antibiotic-resistant genes in the respective strains from different environment within Africa and make a case for specific scientific approach(es) needed to treat the pathogen's infections.

## 2. Materials and Methods

### 2.1. Data Acquisition

The entire one hundred and sixty-one (161) WGS of *H. pylori* from different geographical regions of Africa, isolated from the stomachs of patients from 2012 to 2022, were downloaded from the BV-BRC database [24] for comparative and pangenome analysis. Quality checks were only performed by examining the parameters of the metadata to exclude duplicated and extremely small-size genomes since we were using already assembled genomes in fasta file formats. Genomic features such as the GC content, number of contigs, tRNA, rRNA, CDS, contamination, and completeness were extracted from the final metadata for correlation analyses (Table 1).

### 2.2. Data Analyses

**Figure 1:**
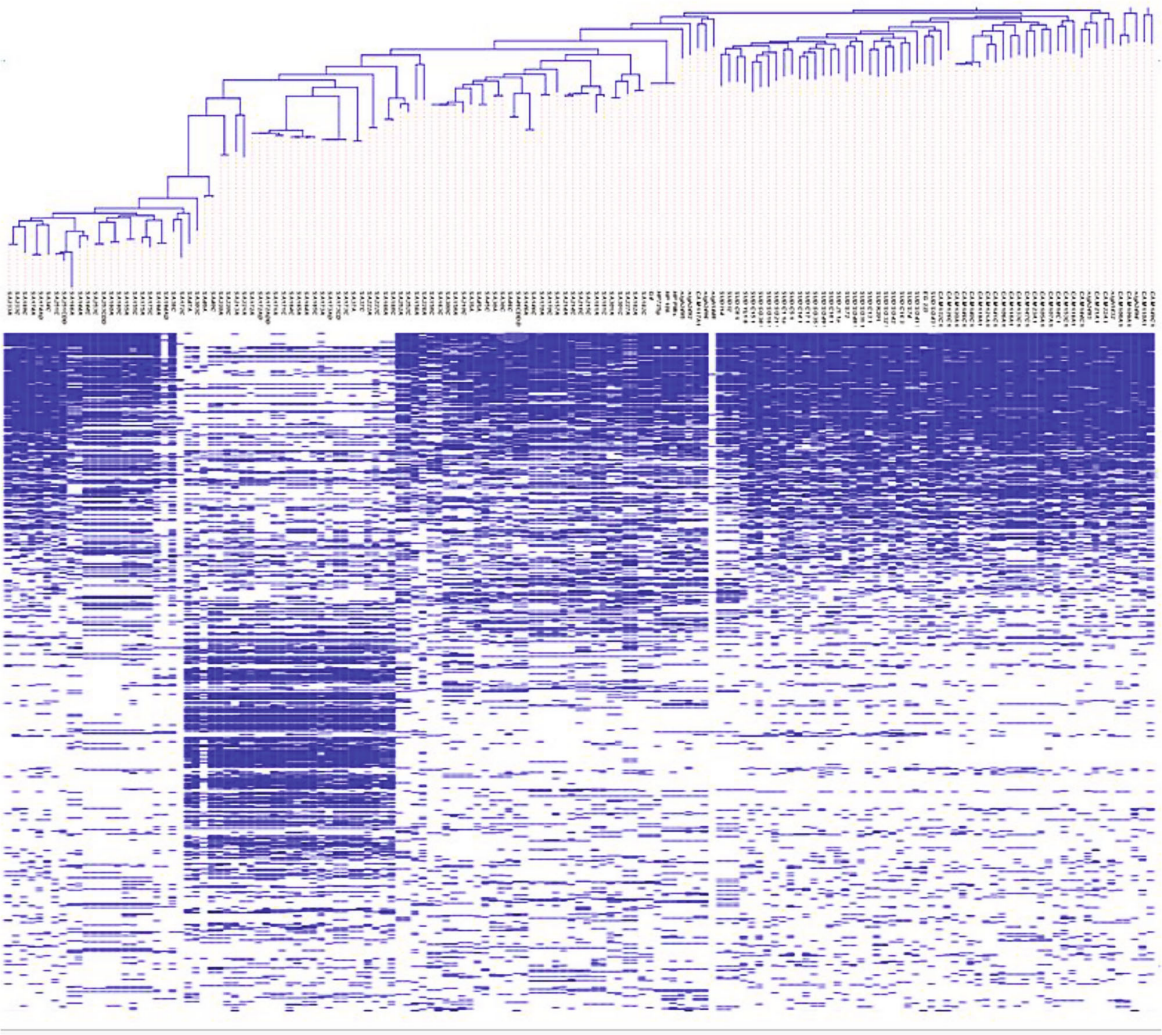
Pangenome matrix illustrating the gene presence and absence in the genomes. Blue and white denote the presence and absence of the particular gene in the respective genomes that were analyzed. Isolates with the same gene at given locus present blue continuous colour, whereas absent genes show a continuous white colour.

**Figure 2:**
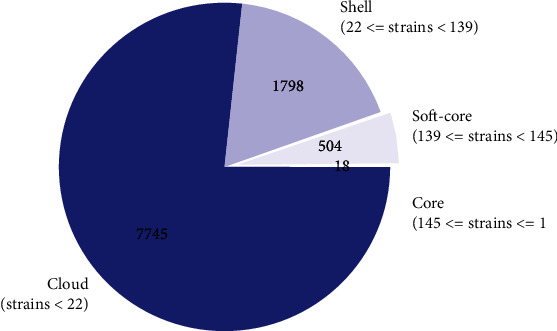
Pangenome pie quantifying the different pan genes. The violet sector shows shell genes, deep blue sector shows cloud genes, white sector shows soft-core genes, and second light violet sector shows hardcore genes.

**Figure 3:**
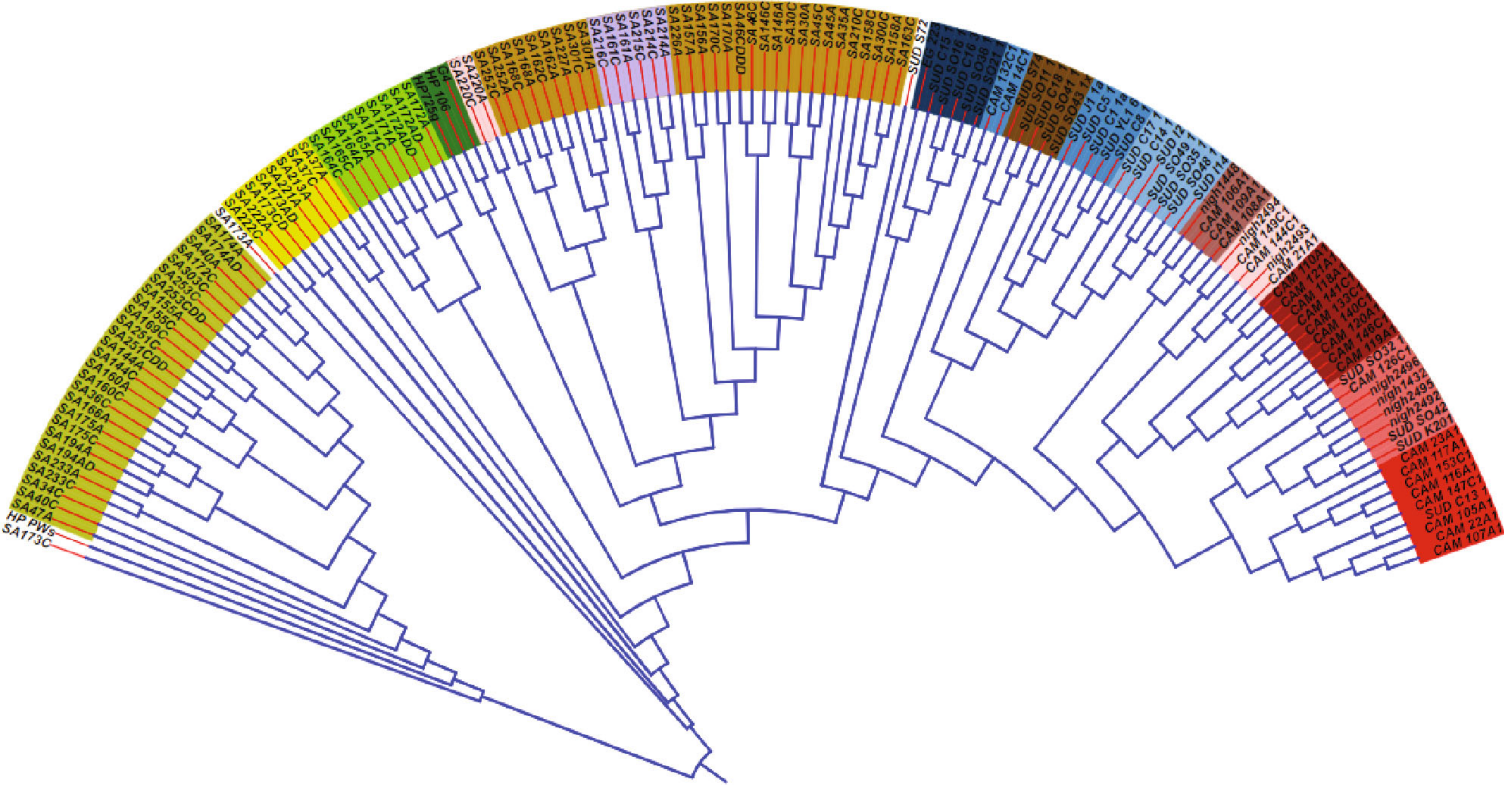
Phylogenetic tree of the isolates generated with iTOLS. Phylogenetic tree showing the evolutionary relationship among strains. The bulk tree is divided into subtrees, coded with colour shades to highlight the distinctions between each subtree and distinct branches relative to the clustering and subclustering which is also colour shade in Figure 4.

**Figure 4:**
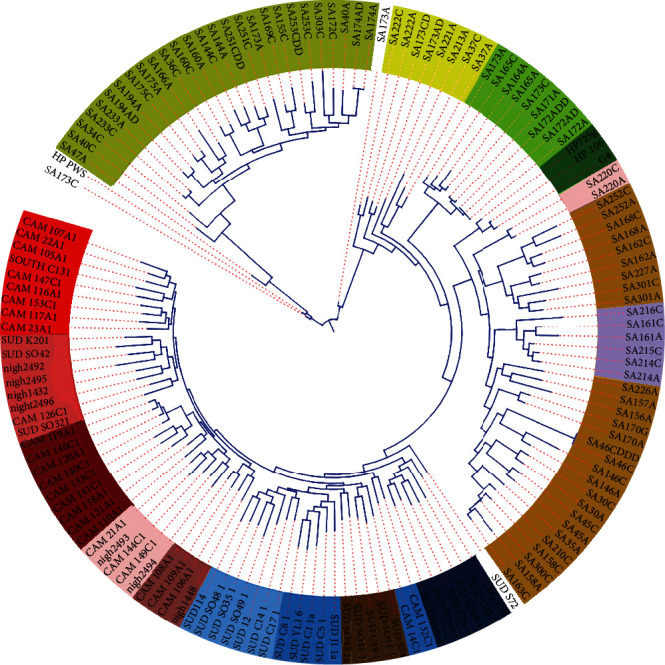
Clustering of the isolates using iTOLS. Strains in unshaded regions did not show any clustering.

**Figure 5:**
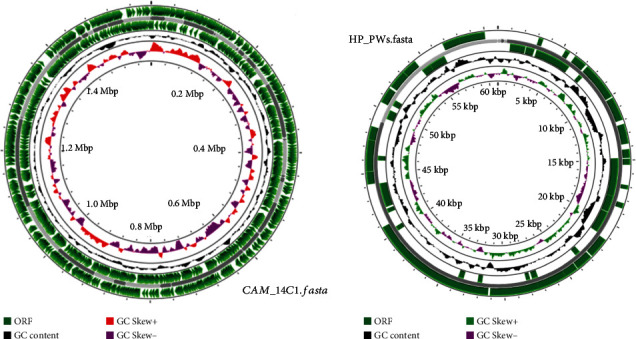
Genome map of overview of selected genomes of *H. pylori* strain. The plot displays the genomes as circular chromosomes. The black outermost and innermost rings indicate the genes which were predicted and annotated using Prokka v.1.14.6, the grey dashes indicate contigs, green rings are the ORFs, and GC skew and GC content are indicated innermost in zigzag rings. The entire genome maps were visualized using fasta files in Proksee (https://proksee.ca/).

**Figure 6:**
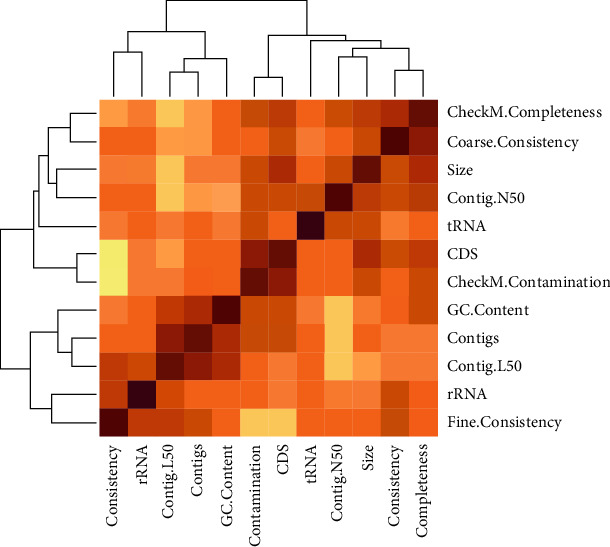
Heatmap comparing the genomic features of the strains generated in RStudio. The pale colours illustrate no correlation, light brown illustrates a weak correlation, deep brown shows a correlation, and white spaces illustrate no correlation.

The final 146 genomes were reannotated using Prokka version 1.14.5 [25]. The *gFF* files from the annotation were used for pangenome analysis using Roary pangenome analysis pipeline (Figures 1 and 2) [26]. Clustering and phylogenetic trees were constructed using Unipro ugene version 48.1, online tool, iTOLS (Figures 3 and 4and Table 2); exploration of the genome's maps for ORFs was generated using Proksee (https://proksee.ca/) (Figure 5), and correlational analyses of the genomic features of the genomes were done using R version 4.3.0 (Figure 6 and Table 3).

**Figure 7:**
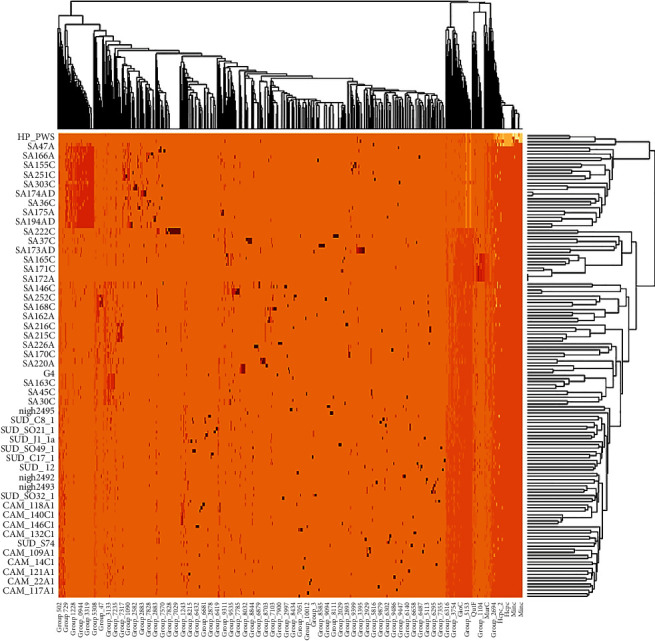
Heatmap showing clustering of pan genes, strains, and gene presence and absence. Deep brown shades show a high concentration of the corresponding genes, light brown shows less concentration of the genes, and white colour shows absence of gene in the corresponding genome.

Statistical and downstream analyses and visualization in this segment were performed by using FastTree version 2.1, Phandango version 1.3.0, and RStudio version 4.3.0 with ggplot2 package (Figure 7).

**Figure 8:**
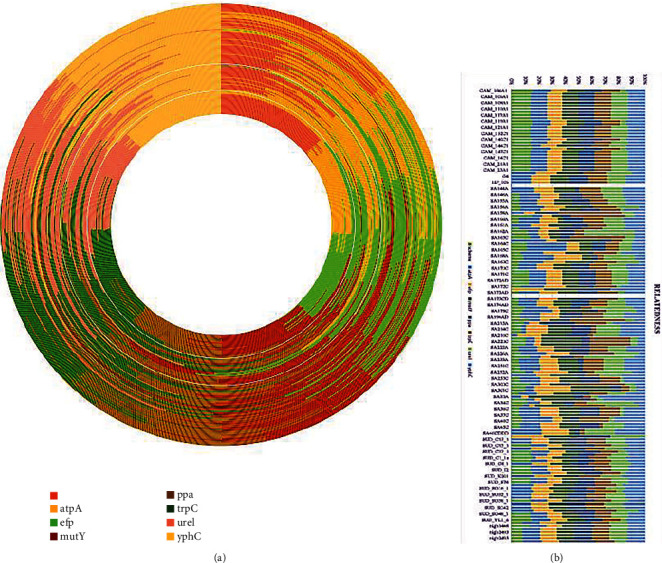
(a) Doughnut and (b) stack bar plots illustrating genetic relatedness of the strains were generated in Google Sheets. Each ring and stack bar plot represents a genome, and the colours represent the housekeeping genes. The length of each colour shows the allelic number or number of alleles in that locus or gene position. The numbers could also be considered as identities. Bars with the same patterns of colours are considered as having the same identity numbers and are therefore related.

Genetic relatedness was assessed first using the Multilocus sequence typing, MLST package version 2.11, in command line to obtain a CSV file of the seven housekeeping genes with their allelic identity numbers. The allelic numbers were extracted using Excel with genomes as row names and the seven housekeeping genes as column headings and the allelic numbers as entries and visualized using Google Sheet's doughnut package and bar plot (Figure 8).

**Figure 9:**
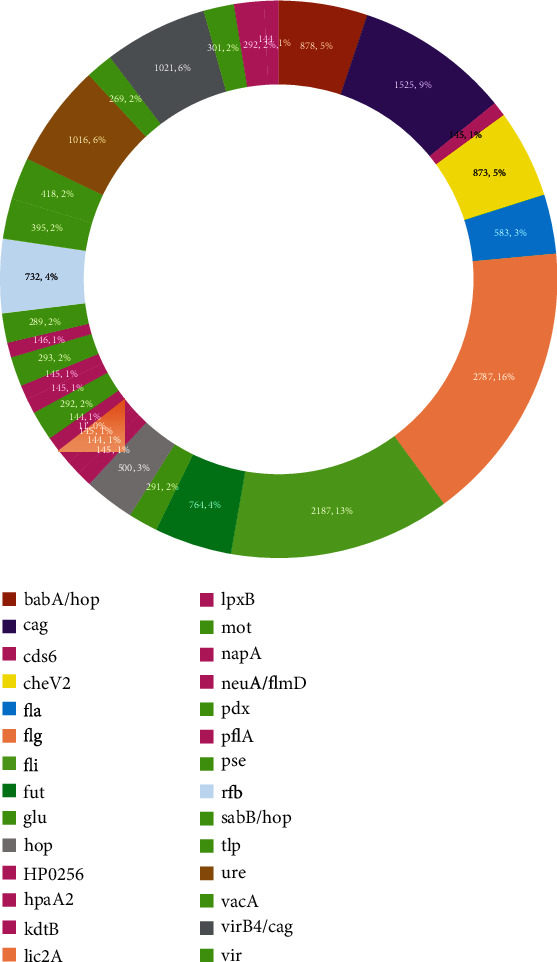
A doughnut plot generated in Google Sheets showing the most predominant virulent genes found in the entire strains.

**Figure 10:**
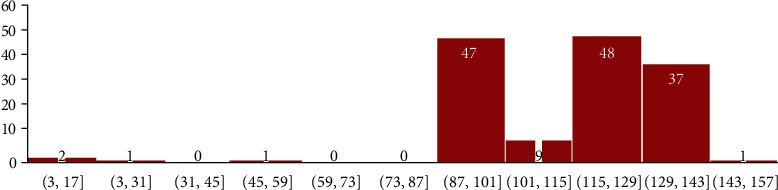
Virulent factor distribution over the strains, generated using Google Sheets.

Ascertainment of virulent and antibiotic-resistant factors of the bacterium was done using Abricate version 2.4 [27]. In detecting the AMR genes and the respective antibiotics, the Abricate software was used with ResFinder as the database. For the assessment of virulent genes, the Abricate was used with the virulent factor database VFDB. Further statistical analyses and visualizations for both domineering virulent genes and distribution were done using Google Sheets and R version 4.3.0 with the ggplot2 package (Figures 9 and 10).

## 3. Results

One hundred and forty-six (146) different *H. pylori* strains isolated from the same source from different countries across Africa and sequenced on the Illumina platform were used in this study. Fifteen (15) genomes were of poor quality and duplicates and were removed.

### 3.1. Analyses of the Genomic Features of the *H. pylori* Bacterium Isolated in Africa

Overall, the genome sizes of the 146 strains ranged from 4841074 KB to 7101918 KB. The GC content for all the genomes was an averagely 38.9%. The number of contigs ranged from 26 and 282 contigs and a corresponding N50 of between 17985 KB and 269337 KB, respectively. The averages of the genomics features have been tabulated in Table 1 with the summarized implications and references.

Table 3 explains in detail the correlations between each pair of the genomic features indicated on the first row and first column of the table. A coefficient of 1 means a strong correlation between the pair, and 0 means no correlation. Positive coefficients mean direct relation and negative means inverse relationships.

### 3.2. Genome Characteristics of the *H. pylori* Strains

In genomics, an ORF (open reading frame) is very important in characterizing a genome because of its potential to be translated into a protein due to the start codon (typically ATG, which codes for the amino acid methionine) and end codons (such as TAA, TAG, or TGA), indicating the termination of protein synthesis. In this segment of the study, we compared factors such as the presence of start and stop codons, the length of the ORF, and the absence of premature stop codons within the sequence of six (6) selected strains from different locations.

### 3.3. Clustering and Phylogenetic Analytics of the *H. pylori* Strains from African Setting

The clustering of bacteria refers to the process of grouping or categorizing bacteria based on their similarities in certain characteristics, such as genetic relatedness, phenotypic traits, or ecological behavior. Clustering methods are widely used in microbial taxonomy, phylogenetics, and metagenomic studies to understand the diversity, evolution, and ecological relationships among bacterial species.

### 3.4. Establishing the Genetic Relatedness of the *H. pylori* Strains from Africa

Determination of genetic relatedness is very important in comparative analytics of bacteria. It establishes the percentage of similar genes shared by two or more pathogens or their strains. According to Hamilton's rule, genetic relatedness predicts the form and frequency of altruistic and competitive behavior, such that organisms are most altruistic and least competitive with those to whom they are most closely related. In related analyses, such pathogens will react to drugs similarly. Figure 8 pictures the relatedness of the *H. pylori* strains.

### 3.5. Exploring the Pangenome Composition of the *H. pylori* Strains from Africa

In this segment, pangenome analysis using the Roary pangenome pipeline was done for the entire collection of genes, genomes, and genetic variations within the *H. pylori* strains. It involved the comparative analysis of multiple genomes to identify and characterize the core genome (genes present in all individuals), accessory genome (genes present in some strains), and unique or strain-specific genes.

Table 2 presents a summary statistic of the pangenome analysis generated with Roary. The table highlights the types of pan genes, the coverage of the genome which harbored the corresponding genes, and the total number of those genes present in the pan genome.

### 3.6. Virulence of *H. pylori* Strains from Ancient Africa

*H. pylori* is known for possessing features that aid it to colonize the human stomach leading to various associated gastric diseases, including gastritis, peptic ulcers, and gastric cancer. The virulence of *H. pylori* is attributed to the presence of certain genes and genetic elements that contribute to its pathogenicity. Here is a comparative analysis of both the well-known virulent genes of novel ones identified in this study.

Each number on the bars indicates the number of strains while the *x*-axis outlines the range of virulent factors they contained. As such, 47 strains recorded between 87 and 101 virulent factors; 9 strains recorded 101-115 virulent factors; 48 strains recorded 115-129 virulent factors while 38 recorded 130 virulent factors and above.

## 4. Discussion

The aim of the study was to explore the diversity of the *H. pylori* strains using its pangenome composition, the distribution of its virulence genes, and the presence of antibiotic resistance genes of the bacterium's isolate from selected regions of Africa. We first explored the genomic features of the isolates and noted that the GC content for all the genomes hoovers around 39% signifying a less stable genome with low melting point in concordance with Behrens' report of average GC of 38.7% with respect to strain 26695 (38.9%) and strain J99 (39.2%) [38]. Other features were averagely normal with little to no correlation between any pair except in a case of CDS and coarse consistency which showed strong positive correlation but reverse in the case of CDS and CheckM contamination. In general, no observable pattern of association was noticed among the genomic features, which could suggest an independent development of each feature from another.

We also explored ORFs of the strains for prediction of potential proteins the bacteria strains may encode, estimate protein function, and examine evolutionary conservation. We found varying proportions of the ORFs, long and short ORFs. For instance, long ORFs are often used to identify candidate protein-coding regions or functional RNA-coding regions in a DNA sequence [39], while short ORFs (sORFs) are used to indicate absence of classical hallmarks of protein-coding genes (both from ncRNAs and mRNAs) but can produce functional peptides [40]. The selected strains showed more sORFs than the long ORFs.

Exploring the diversities among the strains, ten (10) clusters in colour shades were observed. In general, strains from the same geography were clustered together except in a case of the grey-shaded cluster containing strains from Nigeria, Sudan, and Cameroon. We also noted strains from Egypt and Sudan clustering together. However, all the strains from South Africa clustered distinctly with no other strains except for Moroccan strain HPpws. This clustering suggests a localized similarity as against cross-geographical diversities. We also noted a pattern in the clusters that followed the regional boundaries. For instance, a cluster that is dominated by strains from Sudan is swiftly followed by a cluster with Egypt's strains and same pattern with Nigeria and Cameroon and Sudan and Morocco. This pattern may imply proximity as a factor for the spread of the bacterium.

Examining the genomes, over 20 k virulence genes were identified. These genes code proteins whose role relates basically to the *H. pylori* motility, chemotaxis, adherence, acid resistance, and host tissue damage. The genes detected were summarized into 15 major groups in terms of functions [41]; the BabA/hop genes, flg genes, fut genes, ure genes, cag gens, vacA genes, sabA genes, etc. The *H. pylori* flagellates alone constituted 2787 genes making up of 16% of the entire pangenome compared to the other groups. The finding is in tandem with previous report [42] that the flagella are the power hub of the *H. pylori* virulence activities and constitute the large collection of virulence factors in the *H. pylori* pathogen. This perhaps presents a strong clue for further analyses.

Besides the flagellate, other virulent gene observed was the T4SS. The T4SS is known for its association with ulcers and adenocarcinoma [43], found in many species of bacteria [44] and involved in a diversity procedure, including the swapping of genes between adjacent bacteria. The systems typically have at least twelve (12) components [45]. However, in this study, only eight of those components were predicted which is in line with findings of eight (8) classes by [46]. These components have been reported to play tangential role in forming a single working mechanism towards promoting virulence in the host organism [47]. Two of the eight also showed more dominance as compared to the rest of the subgroups. But unlike the flagellates, the T4SS were not uniformly distributed across the genomes. Some isolates from South Africa did not record any of the *cag* genes. This observation is contrary to the findings which reported the coexistence of *cag and vacA* genes in every isolate of *H. pylori* [48]. The observation further suggests that *cag* gene might not be present in all *H. pylori* isolates which strongly suggest that the cag pathogenicity island might be absent in some *H. pylori* isolates.

The *vacA* gene was relatively less observed in all the isolates, an observation that is in line with similar findings elsewhere [49]. The *vacA* gene was more prevalent in the isolates from South Africa, which is consistent with the findings of [50] whose works also observed 90% of the *vacA* gene in South African *H. pylori* gastric patients. However, the *vacA* gene was sparingly present in the Nigerian isolate and completely absent in the Morocco isolates, contrary to previous claims of cohabitation of the genes [48].

In many bacteria species such as *Proteus mirabilis*, *Staphylococcus saprophyticus*, and *H. pylori*, urease enzyme is considered cytoplasmic protein [51] with *ure*A and *ure*B as the main subtypes. The enzyme is the main barrier breaker [2] for the *H. pylori* bacteria surviving in a gastric environment where 99.9% of other bacteria will die. In this study, the urease enzyme of *H. pylori* was found in all the genomes and constituted among the largest gene groups being considered. However, different subtypes of the enzymes were noted in the genomes. Of these, only five of them (*ureA*, *ure*B, *ure*F, *ure*I, and *ure*H) are said to be essential for urease expression and functional activity [52]. The *ure*A and *ure*B are structural genes, and *ure*F, *ure*I, and *ure*H are accessory genes. The structural genes which lack N-terminal were much more observed in the South African genomes while the accessory subtypes noted for being essential for enzyme activity dominated the North African isolates such as the Moroccan genomes.

The lipopolysaccharide (LPS) group was notably present in all the genomes. LPS forms the major proteins on the surface of most Gram-negative bacteria including *H. pylori* and is composed of lipid A-core and the O-antigen polysaccharide. The LPS group was found to be comprised primarily of the Lewis antigens (*kdt*B) and lipopolysaccharide core biosynthesis protein, lipopolysaccharide 12-glucosyltransferase (*rfa*J), fructosyltransferase lipopolysaccharide Lewis antigens (*fut*C), and lipopolysaccharide heptosyltransferase *(rfa*C) noticeably found in all the isolates. Literature has indicated that the quantity and location of Lewis antigens in the LPS differ among *H. pylori* isolates, indicating an adaptation to the host [53].

The adhesins (*bab* genes) were among the least observed genes in the entire study with a total implying that some genomes did not record the genes such. It must be emphasized that the adhesins had a poor hit with the Abricate software, which might have accounted for the low observation of the gene among the isolates. The adhesin group helps in initiating virulence by influencing attachment of the bacteria to the gastric membrane cells [54]. Comparatively, the adhesins dominated in isolates from Morocco than the rest of the other isolates.

The difficulty in treating the *H. pylori* pathogen from among human race is the upsurge of its virulence genes [55]. In this study, all the isolates profiled using the Abricate software have each returned more than 60 virulence genes in nominal counts. However, the distribution of the genes among isolates varied. First, there was a variation between the number of virulence genes recorded per isolate with the Moroccan genome recording the highest number of genes (147 virulence genes) same to the findings by [56] who identified 147 virulence genes. The South African isolate SA50 recorded the least number of genes (60 virulence genes). This suggests that *H. pylori*isolates have unproportionate number of virulence genes. Interestingly, the genome size of the Moroccan isolate was relatively larger (1648327 bp) than the SA50 isolate's size (1600214 bp) but smaller than the Moroccan isolate mor_G4. This might also suggest that *H. pylori* genome sizes and number of virulence genes contained in them are indirectly proportional.

Conspicuously missing in the virulent factors is the *dupA* gene. This gene was the first genetic factor of *H. pylori* detected to be associated with a varying susceptibility to duodenal ulcers and gastric cancers [57]. It has since been considered as a disease-specific virulence marker in some parts of the world [58]. However, the gene could not be detected in all the 146 isolates from the selected regions of Africa in our study. But studies have already established that the prevalence of the *dupA* gene is pointedly higher in strains isolated from duodenal ulcer patients than with gastric cancer patients (42% vs. 9% on average), regardless of nationality [59]. We therefore relate our findings to the isolation source (stomach) as the reason for the absence of the *dupA* gene.

In Africa, the commonest approach to treating the *H. pylori* bacteria from infected persons is the use of antibiotics such as clarithromycin, tinidazole, metronidazole, amoxicillin, and levofloxacin. However, heteroresistance of the bacterium in a case of clarithromycin and levofloxacin has been reported [60]. A case in point is the *vacA* gene which is much associated with clarithromycin and tinidazole resistance and the *cag*A gene which is also associated with tinidazole resistance [61]. Strong association between amoxicillin resistance and the *hop* gene of the bacterium has been reported [62]. Ureases have been reported to be strongly associated with metronidazole resistance in the bacterium [63], while *babA* and *babB* genes have been implicated in the *H. pylori* erythromycin resistance [64]. The *pbp1A* gene has been reported to be associated with amoxicillin resistance.

Other significant genes, which have been reported of their resistance against known antibiotics, were also identified in some of the genomes. The *Oip* gene, which formed part of the adhesins and is observed in all the genomes, is reported of its resistance against streptomycin. Some unique genes which were observed in the isolates including *23S rrn*, *rdxA*, *frxA*, *gyrA*, *rpoB*, *pbp-1A*, and *16S rrn* genes were resistant to macrolides, metronidazole, metronidazole, quinolones, rifamycin, amoxicillin, and tetracycline, respectively [65].

Equally very important finding which this study explored on is genetic relatedness, which is being described as the probability that the alleles between two organisms and in this case *H. pylori* bacteria are identical by descent. This is so significant in assessing pathogens given that genetically related organisms react to drugs similarly. The isolates from North Africa such as the mor_G4, mor_Hp_pws, Mor_Hp106, and Mor_Hp725g showed similar allele numbers of all the seven housekeeping genes and may suggest some level of relatedness. Isolates SA158A, SA163A, SA163C, SA210A, SA210C, SA300A, SA300C, SA31C, and SA158C from South Africa were found to cluster together with near uniform allele numbers which also suggest some relatedness. However, the rest of the isolates rather showed nonuniform allele numbers and could mean unrelatedness. Interestingly, the relatedness as determined using the MLST did not correlate with the genes identified in those isolates. For instance, the Moroccan isolates though suggested genetic relatedness, the genes hit by Abricate in each of those isolates, were not the same. This could mean that isolates may have similar allele numbers yet contain not exact number or types of genes, which corroborates the previous findings that genetically related isolates may not be the same due to recombination and horizontal gene transfer [66].

Another layer we explored in this work is homology which in a genetic sense is a resemblance of the structure, physiology, or development of different species of organism dependent on their descent from a common evolutionary ancestor [67]. As a result, it was crucial to assess the isolates in terms of homology, similarity, descent, and shared family tree to determine their variabilities. Pangenome matrix findings corroborated to a larger extent the genetic relatedness of the 146 isolates. Isolates that clustered together exhibited some level of relatedness implying that those isolates descended from the same ancestry. Again, from the pangenome analysis, hardcore genes (223) were recorded which suggest the presence of homologous families among the isolates of 50% shared relationship [68]. Besides the hardcore, the soft-core genes (333) were identified, which also suggests the presence of a homologous family that share 90% identity among the isolates. However, the shell genome of about 1233 genes suggests that 50% of the isolates in this study have undergone evolutionary changes that is gene gain and gene fixation [69] and the cloud pangenome also referred to as accessory genome that contains genes only found in a single isolate was also observed. About 3450 genes formed the cloud genome in the study, a finding that may suggest a gene families developed through ecological adaptation.

## 5. Summary

Our findings revealed that *H. pylori* from the African subregion harbors a multitude of virulence genes in its genome quantifying over 20,000 from 146 isolates. With an average of 92 virulent genes, 19 out of the 146 isolates contained between 130 and 148 virulent genes and were considered the most virulent. Quantifying the individual virulent factors , the flagellates were more in relation to the other factors especially the cag and vac genes, reaffirming their grieve contribution to the disease onset. DupA gene which has been considered as a disease-specific virulent marker in some parts of the world could not be detected in all the genomes used in this study.

In comparing the ORFs, all the selected strains showed more sORFs than the long ORFs. The SA37C produced more of the short ORFs and zero long ORF, while CAM-21A-1 and SUD-SO35-1 both returned fewer sORFs. On the contrary, the CAM-21A-1 and SUD_SO35_1 equally produced longer ORFs than the rest.

Multilocus sequence typing analyses of the seven housekeeping genes *(atpA*, *efp*, *mutY*, *ppa*, *trpC*, *ureI*, and *yphC*) strongly suggest that majority of *H. pylori* isolates (95%) from Africa are diverse. This outcome may imply that two or more *H. pylori* isolates taken from the same geographical location in Africa stand 95% chance of being unrelated. This further strengthens the call on individualized treatment of *H. pylori* infections.

With respect to antibiotic resistance, at least two (2) AMR genes and at most seven (7) were found in all the isolates, an indication of varied responses of each isolate to a given antibiotic. However, the gene which showed resistance to clarithromycin, *23SrRNA*, and that of the gene that also showed resistance to amoxicillin, *pbp2*, were both found in all the isolates. This could mean that these two antibiotic potencies against *H. pylori* might be approaching obsoletion. Other interesting pattern observed was that the four (4) isolates from North Africa which earlier showed genetic similarity with the ***MLST*** algorithm also returned the same number of AMR genes and same observed with south African isolate SA30A and SA30C. This could literally suggest that isolates with same locus profile might be having same resistance pattern against antibiotics.

This study found both virulence and AMR genes in all *H. pylori* strains in all the selected geographies around Africa with differing quantities. MLST, pangenome, and ORF analyses showed disparities among the isolates. This in general could imply diversities in terms of genetics, evolution, and protein productions. Therefore, generic administration of antibiotics such as clarithromycin, amoxicillin, and erythromycin as treatment method for H. pylori infections in the African subregion could be contributing to the spread of the bacterium's antibiotic resistance.

## Figures and Tables

**Table 1:** Averages of genomic features of *H. pylori* and their implications.

Genome feature	Average	Implication	References
Contigs	61.5	Good number of overlapping clones	[28]
G-size	1627587.8	More genes, more and longer introns, and more transposable elements	[29]
GC content	38.9	Unstable genome with low melting point	[30]
Contig L50	8.1	Total number of chromosomes, organelle genomes, and plasmids in the assembly	[31]
Contig N50	80187.7	Assess the contiguity of an assembly	[32]
tRNA	35.5	Normal	[33]
rRNA	2.4	Overloaded the column	[34]
CDS	1660.1	Corresponds to amino acids in a protein	[35]
Coarse consist	99.8	The genome qualities are good	[36]
Fine consist	99.0	The genome qualities are good	[36]
CheckM contamination	2.3	The genome qualities are good	[37]
CheckM completeness	99.9	The genome qualities are good	[37]

**Table 2:** Summary statistics, pangenome.

Pan_gene	Coverage	Num
Core	99% ≤ strains ≤ 100%	18
Softcore	95% ≤ strains < 99%	504
Shell	15% ≤ strains < 95%	1798
Cloud	0% ≤ strains < 15%	7745
Total	0% ≤ strains ≤ 100%	10065

**Table 3:** Correlation coefficients of the genomic features of the strains.

	Contigs	Size	GC content	Contig L50	Contig N50	tRNA	rRNA	CDS	Coarse consistency	Fine consistency	CheckM contamination	CheckM completeness
Contigs	1	-0.1	0.5	0.7	-0.5	-0.1	0	0.1	-0.3	0	0.1	-0.3
Size	-0.1	1	-0.1	-0.5	0.3	0.1	-0.2	0.6	0.2	-0.1	0.2	0.5
GC content	0.5	-0.1	1	0.4	-0.5	-0.2	0	0.1	0	-0.1	0.1	0.1
Contig L50	0.7	-0.5	0.4	1	-0.7	-0.2	0.1	-0.3	-0.3	0.2	-0.1	-0.4
Contig N50	-0.5	0.3	-0.5	-0.7	1	0.1	-0.2	0.1	0.1	-0.1	0	0.2
tRNA	-0.1	0.1	-0.2	-0.2	0.1	1	-0.1	0	-0.2	-0.2	0.1	0
rRNA	0	-0.2	0	0.1	-0.2	-0.1	1	-0.2	0.1	0.2	-0.1	-0.1
CDS	0.1	0.6	0.1	-0.3	0.1	0	-0.2	1	0.2	-0.7	0.8	0.5
Coarse consistency	-0.3	0.2	0	-0.3	0.1	-0.2	0.1	0.2	1	0.1	0.1	0.6
Fine consistency	0	-0.1	-0.1	0.2	-0.1	-0.2	0.2	-0.7	0.1	1	-0.9	-0.2
CheckM contamination	0.1	0.2	0.1	-0.1	0	0.1	-0.1	0.8	0.1	-0.9	1	0.3
CheckM completeness	-0.3	0.5	0.1	-0.4	0.2	0	-0.1	0.5	0.6	-0.2	0.3	1

## Data Availability

All the data used in this study can be found on the Bacterial and Viral Bioinformatics Resource Center (BV-BRC) database (https://www.bv-brc.org) with specific link: https://www.bv-brc.org/view/Taxonomy/210#view_tab=genomes~~~~~~~~~^~^~^~^~~~~~~~~~~~amp;filter=and(eq(geographic_group,“Africa”),eq(genome_status,“WGS”),eq(genome_quality,“Good”)).
